# Prospective comparison of circumferential and longitudinal strain in asymptomatic children with single left ventricle, single right ventricle and normal hearts

**DOI:** 10.1186/1532-429X-16-S1-P111

**Published:** 2014-01-16

**Authors:** Cory Noel, Ramkumar Krishnamurthy, Amol Pednekar, David Chu, Rajesh Krishnamurthy

**Affiliations:** 1 Radiology, Texas Children's Hospital, Houston, Texas, USA; 2 Pediatrics, Baylor College of Medicine, Houston, Texas, USA; 3 Philips Healthcare, Houston, Texas, USA

## Background

Ventricular dysfunction in patients with a single right ventricle (SRV) or a single left ventricle (SLV) is a known risk factor for morbidity and mortality. However, the differences in SRV and SLV function remain poorly understood, with only a few studies performed1-3. In this study, we measure the strain using cardiac MRI and perform comprehensive comparison of the global and regional strain in both the circumferential (εcc) and longitudinal (εL) dimension. Purpose: In normal subjects and asymptomatic patients with SLV and SRV after total cavopulmonary connection (TCPC), compare: 1) Global εcc and εL strain, 2) Regional circumferential and longitudinal strains at free wall (εcc-free, εL-free) and septum (εcc-sept, εL-sept), 3) εcc and εL across the ventricle from apex to base.

## Methods

We performed a prospective analysis of 18 subjects (7 normal age: 11.8 +/- 3; 6 SRV age: 11.4 +/- 2.3; 5 SLV age: 12.7 +/- 4.2). Acquisition Protocol: Strain information was acquired at three short axis slices at basal, mid-cavity, and apical locations in all 18 subjects in a 1.5T MRI scanner (Philips Acheiva) using: a) Complementary Spatial Modulation of Magnetization (CSPAMM)4 images: Used for generating εcc; and b) Fast-Strain Encoded (fSENC)5 images: Used for generating εL. Data Analysis: εcc and εL across all cardiac phases and slices were calculated from SAX slices using DiagnosoftTM. The ventricular regions at each slice were assigned based upon the AHA 16 segment model. εcc-sept, εL-sept, εcc-free, and εL-free were also calculated for each slice and compared.

## Results

**Figure 2:**
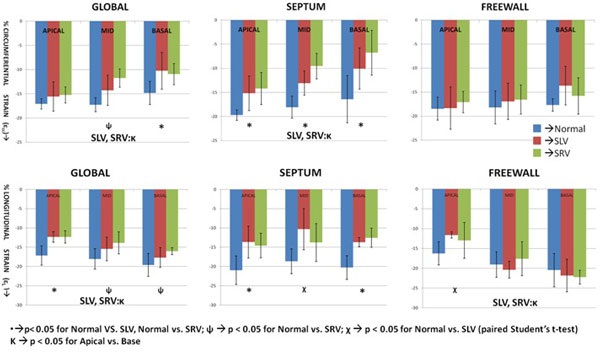
**Bar plots showing the longitudinal (ε_L_) and circumferential (ε_cc) _strain values in a pediatric population with systemic single ventricles**. We demonstrate a significant reduction in both ε_L _and ε_cc. _The septum is the most affected with negligible differences observed in the free wall. Also, there is a significant difference observed from apex to base globally for both the single systemic ventricle patients, while the free wall ε_L _shows a significant increase.

Compared to normals, 1. Significant reduction seen in global εcc at mid and basal locations of both SLV and SRV patients (Figure 2). 2. Significant reduction seen in global εL in apical locations of SLV and SRV patients. 3. SV groups exhibited significant reduction in septal strain. This was significantly higher than the reduction in global strain. 4. εcc-sept and εl-free significantly changed from apex of the ventricle to the base, which is closer in proximity to the hypoplastic chamber.

## Conclusions

Strain values of SLV and SRV demonstrate significant differences compared to normal subjects. Septal circumferential strain is significantly reduced in single ventricle patients while the free wall strain is normal. Circumferential strain of the SV progressively reduces from the apex to the base, suggesting a deleterious effect of the hypoplastic chamber connected to the base.

## Funding

None.

**Figure 1:**
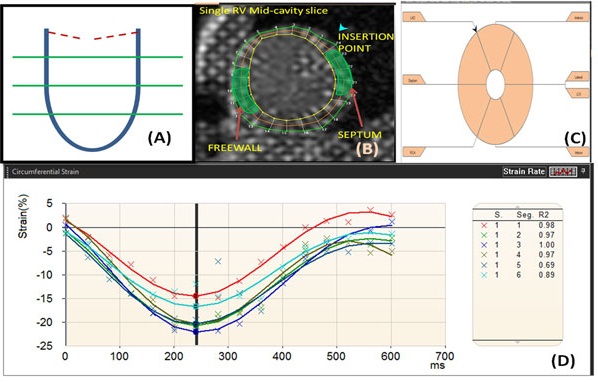
**Schematic depicting the acquisition of strain curves from short axis images**. (A) Three short axis slices images (CSPAMM and fSENC) at basal, mid-cavity and apical locations of the systemic ventricle were obtained. (B, C and D) They were post-processed to obtain localized strain curves. Longitudinal and circumferential strain were obtained as per AHA 16 model guidelines by acquiring both the CSPAMM and fSENC images at the same slice locations respectively.
